# Statistical Learning of Large-Scale Genetic Data: How to Run a Genome-Wide Association Study of Gene-Expression Data Using the 1000 Genomes Project Data

**DOI:** 10.1007/s12561-023-09375-9

**Published:** 2023-07-01

**Authors:** Anton Sugolov, Eric Emmenegger, Andrew D. Paterson, Lei Sun

**Affiliations:** 1 Department of Mathematics,Faculty of Arts and Sciences, University of Toronto, Toronto, Canada; 2 Department of Mechanical and Industrial Engineering, University of Toronto, Toronto, Canada; 3 Program in Genetics & Genome Biology The Hospital for Sick Children, University of Toronto, Toronto, ON Canada; 4 Dalla Lana School of Public Health, University of Toronto, Toronto, Canada; 5 Department of Statistical Sciences, Faculty of Arts and Sciences, Dalla Lana School of Public Health, University of Toronto, Toronto, Canada

**Keywords:** 1000 Genomes Project, Data Visualization, Genome-wide Association Study, Gene Expression, Hands-on Experience, Large-scale Data Analysis, Multiple Hypothesis Testing, Open Resource, Reproducible Research

## Abstract

Teaching statistics through engaging applications to contemporary large-scale datasets is essential to attracting students to the field. To this end, we developed a hands-on, week-long workshop for senior high-school or junior undergraduate students, without prior knowledge in statistical genetics but with some basic knowledge in data science, to conduct their own genome-wide association study (GWAS). The GWAS was performed for open source gene expression data, using publicly available human genetics data. Assisted by a detailed instruction manual, students were able to obtain $\sim$1.4 million p-values from a real scientific study, within several days. This early motivation kept students engaged in learning the theories that support their results, including regression, data visualization, results interpretation, and large-scale multiple hypothesis testing. To further their learning motivation by emphasizing the personal connection to this type of data analysis, students were encouraged to make short presentations about how GWAS has provided insights into the genetic basis of diseases that are present in their friends or families. The appended open source, step-by-step instruction manual includes descriptions of the datasets used, the software needed, and results from the workshop. Additionally, scripts used in the workshop are archived on Github and Zenodo to further enhance reproducible research and training.

## Introduction

The overarching goal of this project is providing an example of engaging education in statistics to attract senior high-school or undergraduate students to the field, who will eventually grow and mature as competent data scientists. To achieve this goal, we designed a week-long workshop that provides students contextual, immersed, and hands-on learning experience in data science, using publicly available, contemporary datasets.

We chose genetic data as the domain knowledge because they are complex, large-scale, high-dimensional, and practically important [39]. Although we do not expect nor want all students to continue their studies in statistical genetics, at the end of the workshop we expect students to (a) know about the variations in the human genome and the structure of the human population, (b) put into use their statistical knowledge by working with the 1000 Genomes Project (1 KG) data [2], and (c) deepen their statistical understanding in areas including confounding [15, 23], heterogeneity [21, 22], using principle component analysis to capture population structure [1, 34, 38], multiple hypothesis testing [20, 41], results interpretation and data visualization [9, 24], and reproducible research [19, 32].

Although in this application we focused on genetic data, computational software is important in many areas of large-scale data science, including for example astrostatistics, engineering and manufacturing data management, health data analytics, quantitative finance, and social network modeling and analysis. We highlight that, while application-specific considerations (e.g., domain-specific data quality control procedures) are important, the key statistical concepts introduced in this workshop are useful to analyze data from many domains other than genetic data. For example, multivariate linear regression is the building block for many applications that involve model fitting and statistical inference. Multiple hypothesis testing adjustment is necessary for any large-scale data analysis to prevent overfitting and p-hacking. Principal component analysis is a dimension-reduction technique popular in many data science fields. Finally, data visualization is increasingly recognized as an integral part of good data science practice.

In the last 15 years, genome-wide association studies (GWAS) have become a highly efficient way to identify genetic variants associated with traits and diseases [26, 29, 44, 46]. The typical method involves testing millions of bi-allelic single nucleotide polymorphisms (SNPs), one-at-a-time for association with an outcome (e.g., the continuous blood pressure or the binary trait of high blood pressure) using either linear or logistic multivariate regression, and more recently generalized linear mixed-effect models [13, 51]. Although the commonly used statistical models are relatively simple for each SNP, the main challenge relates to the size of the human genome and the number of SNPs. For example, in imputed genetic data from the UK Biobank [5, 16], about 10 million SNPs are typically analyzed. Additionally, prior to association testing, several (domain-specific) quality control (QC) steps are necessary to restrict the analysis to SNPs and individuals with high quality data [30].

Most individual-level genome-wide SNP data is not publicly available due to privacy [27]. We chose to illustrate GWAS using publicly available trait and genetic data from the 1000 Genomes project, in which participants consented to their data being made freely available [12]. Due to the small sample size available (about 1000 in total with 88 Yoruban and 102 Utah individuals, small relative to 1,344,840, the number of SNPs analyzed), we chose a trait that is known to be strongly associated with some SNPs with large genetic effects. This way, there would be sufficient power to detect the association with the small sample size; the remaining SNPs serve as negative controls and demonstrate issues pertinent to large-scale multiple hypothesis testing.

There is a wide variation in the level of gene expression in a specific tissue or cell, and much of this variation is influenced by SNPs near to a specific gene. We used an example from earlier literature to illustrate the identification of genetic variation associated with the level of expression of the gene named Endoplasmic Reticulum Aminopeptidase 2 (*ERAP2*) [11]. The ERAP2 gene expression levels were measured in peripheral blood B cell lines in Utah residents with European ancestry, and Yoruba people from Ibadan, Nigeria from the 1000 Genomes Project [40]. The project is a publicly available catalogue of individual-level human genetic variation,^1^ constructed by measuring genetic variation with an array of technologies in multiple populations around the world [2].

The workshop is designed to be executed with a 4–5 day period. The mornings can be used for the more traditional teaching modus operandi via lectures, while the afternoons may be dedicated to the hands-on component with sufficient Teaching Assistant (TA) support. The student-TA ratio could range from 1–5 to 1–10, depending on the readiness of the student cohort. The last 2–3 h of the workshop is recommended for a general discussion and obtaining feedback from the students, and ideally including short student presentations; see Sect. 2.5.

## Methods

First and foremost, the workshop provides extensive hands-on experience in conducting, summarizing and interpreting a genome-wide association study to senior high-school students or junior undergraduate students with basic knowledge in data science. The hands-on experience includes using R [28], running PLINK v1.9 [36] which is specific to the GWAS domain, and working with large-scale data. A detailed manual is attached as an Appendix. The most updated version, including an analogue of commands for PLINK v2 [8], is openly accessible.^2^

Additionally, the workshop has the more traditional teaching and learning component through (interactive) lectures, covering complementary topics in genetics and statistics. We have made the lecture notes openly accessible.^3^

### Datasets

In total, 190 individuals and 1,344,840 bi-allelic SNPs from the 1000 Genomes Project [2, 40] passing quality control from The Centre for Applied Genomic (TCAG)^4^

Quality control is a significant component of conducting a proper GWAS [30]. However, in-depth QC is domain-specific and time-consuming, not suitable for the purpose of this workshop. We thus provides a set of good quality data while emphasizing the importance of QC, so that the participating students could successfully carry out a preliminary GWAS within the first two days of the workshop and obtain $\sim$1.4 million p-values from a real scientific study. We note that this early success is critical to keeping the students engaged and motivated to learn the theories that support their empirical results.

[11] identified that the expression of the gene *ERAP2* had strong genetic association in HapMap 3 individuals [25], many of which overlapped with the 1 KG individuals. Gene expressions of *ERAP2* measured in peripheral blood B cell lines were first extracted from Array Express [31, 43], then matched to the IDs of 1 KG individuals, and finally formatted for PLINK; see Appendix 1. The two largest 1 KG sub-populations are Yoruban individuals in Ibadan, Nigeria (YRI), and Utah residents (CEPH, Centre d’Etude du Polymorphisme Humain) with Northern and Western European ancestry (CEU). In total, 91 YRI individuals and 104 CEU individuals matched between an independent subset of the 1 KG with no family relations and HapMap 3 datasets, and these genetic unrelated individuals were used for the workshop purpose.

Using principal component analysis (PCA) of PLINK v1.9 [36], three and two outliers were removed, respectively from the YRI and CEU samples. Thus, the final GWAS analysis was restricted to 88 YRI individuals and 102 CEU individuals, and their genetic data of 1,344,840, bi-allelic SNPs. The basic PCA analysis pipeline is provided in the appended manual and could be part of the workshop if time permits.

### Software

An introduction to PLINK (v1.90 beta 6.24) [35] is necessary for the purpose of this GWAS workshop. PLINK is a command line toolkit for performing the GWAS computation efficiently, giving students hands-on experience with the most popular software used in the ongoing GWAS research. The analysis pipeline was originally implemented with PLINK v1.9 [36] but equivalent commands for PLINK v2 [8] are also provided in a separate manual.

Depending on the readiness of the student cohort (and length of the workshop), a brief introduction to using R (v4.1.0) [37] could be also part of the workshop; open-resource R introduction materials abound.^5^ The installation and use of R packages such as "qqman" [45], "ggplot2" [48] and "hexbin" [7] introduce students to effective data visualization, a core component of interpreting GWAS results. Included in the open source manual is also a brief introduction to an (optional) use of the UNIX environment.

### Overview of the Workshop Content

We summarize the main steps of running a GWAS of the gene expression data of *ERAP2*, using the 88 YRI individuals and their 1,344,840 SNP data of the 1000 Genomes Project (i.e., the YRI GWAS); GWAS is often performed separately for each population [34], as trait distribution and SNP frequency may differ between populations.

We refer the readers to the open source manual and scripts for additional details, which include further analyses (i.e., the CEU GWAS of the 102 CEU individuals and their SNP data) that could be reproduced using the step-by-step instructions. In the analyzed sample, additional PCA may be conducted to capture fine-scale population structure [38]; see Sect. 3 of the appended manual on population stratification.

1. **Prepare the datasets**. Extract the cleaned 1 KG SNP data into a separate analysis-specific directory.
  First, students should specify the phenotype of interest and remove individuals who are not needed for the YRI GWAS. Students achieve these with the –pheno and –prune PLINK commands respectively; for additional details see the section named ‘Standard data input’ of the PLINK v1.9^6^ orPLINK v2.0^7^ documentation.
  Second, students remove rare SNPs (e.g., with a minor allele frequency (MAF) less than 5%) and the sex chromosomes from the analysis using the –maf 0.05 and –chr 1-22 flags, respectively.^8^ (The 1000 Genomes data quality control performed by [40] does not include a MAF-based QC step.)
  Third, we note that Hardy-Weinberg equilibrium is typically part of the QC procedure (using –hardy), as severe departure from HWE is usually an indication of genotyping error [14, 30]. However, HWE is a complex phenomenon and HWE QC criterion is unclear [50]. Thus, the workshop analysis did not include a test of HWE, but we note that HWE should be evaluated for any significant SNPs. Additionally, students should only analyze the autosomal common SNPs, as identifying associations on the sex chromosomes  [10, 47] and analyzing rare SNPs [17] requires more intricate methods beyond the scope of the workshop.
  Lastly, for computational reasons, students create binary files from this dataset with –make-bed. The .bim, .bed, .fam file types should be generated and named after ERAP2_YRI. Students should verify that the parameters they have entered are correct by viewing the .log file.
2. **Run the association analysis**. Since gene expression data is continuous, students should specify a linear regression, with PLINK v1.9 command –linear or PLINK v2.0 command –glm. This evaluates the association between the gene expression and each SNP, also known as the expression quantitative trait loci (eQTL) analysis.
  Association analysis often includes covariates to avoid spurious associations from confounding. The sexes of the individuals are included in the dataset, so students may include this covariate in the eQTL GWAS analysis using –linear sex in PLINK v1.9 or –glm sex in PLINK v2.
3. **Post-association analysis and results interpretation**. The association results can be sorted with sort.R, which also generates a file with the top 50 most significant SNPs. The genome-wide results may be plotted and interpreted, which we explain with examples in the next section; also see the appended manual for additional details. Using appropriate QC steps, including the MAF filtering, prevents NA results in the output in principle. However, to be cautious the NA_removal.R script may be used to identify and remove NA results from the follow-up data visualization analyses. Hardy-Weinberg equilibrium may be checked for the top SNP using –hardy.

### A Highlight: Multiple Hypothesis Testing and Data Visualization

During the workshop, students are introduced to the multiple testing problem in GWAS through the morning lectures. Although the concept of multiple hypothesis testing, and its (theoretical) connection with ‘p-values being Unif(0,1) distributed under the null’, is covered in most introduction courses to statistics, student’s understanding and appreciation of this concept is often lacking, in part due to the traditional emphasis on identifying variables with p-values meeting some significance criterion, as opposed to exploring the whole distribution. This, in part, is a result from a lack of hands-on experience with large-scale real data analysis,

**Fig. 1:**
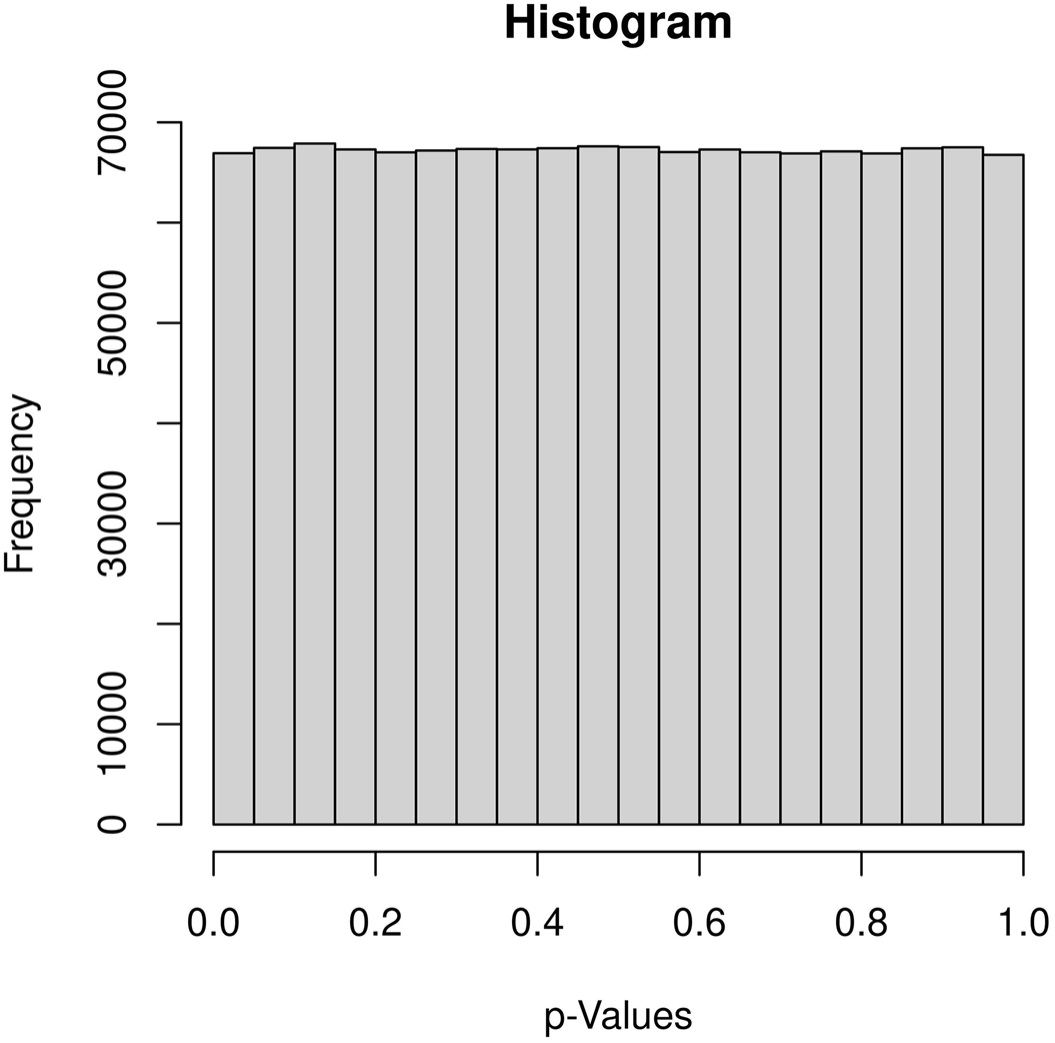
Histogram of the 1,344,840 p-values from the YRI GWAS of the gene expression of *ERAP2*, obtained using the workshop materials. The histogram is close to Unif(0, 1), the expected distribution of p-values under the null of no association

With close to 1.4 million p-values obtained from a real GWAS, students realize that many SNPs (close to 70,000 in fact) are ‘significant’ if the traditional $\alpha = 0.05$ type I error threshold were used. However, the histogram of p-values in Fig. 1 shows an empirical distribution close to Unif(0,1), the distribution expected under the null hypothesis of no association. This is expected for a typical GWAS, as unless the trait is polygenic (i.e., with a large number of contributing SNPs) *and* the sample size is very large, most of the SNPs are not expected to be associated with the trait or their associations are not detectable [18, 49].

**Fig. 2:**
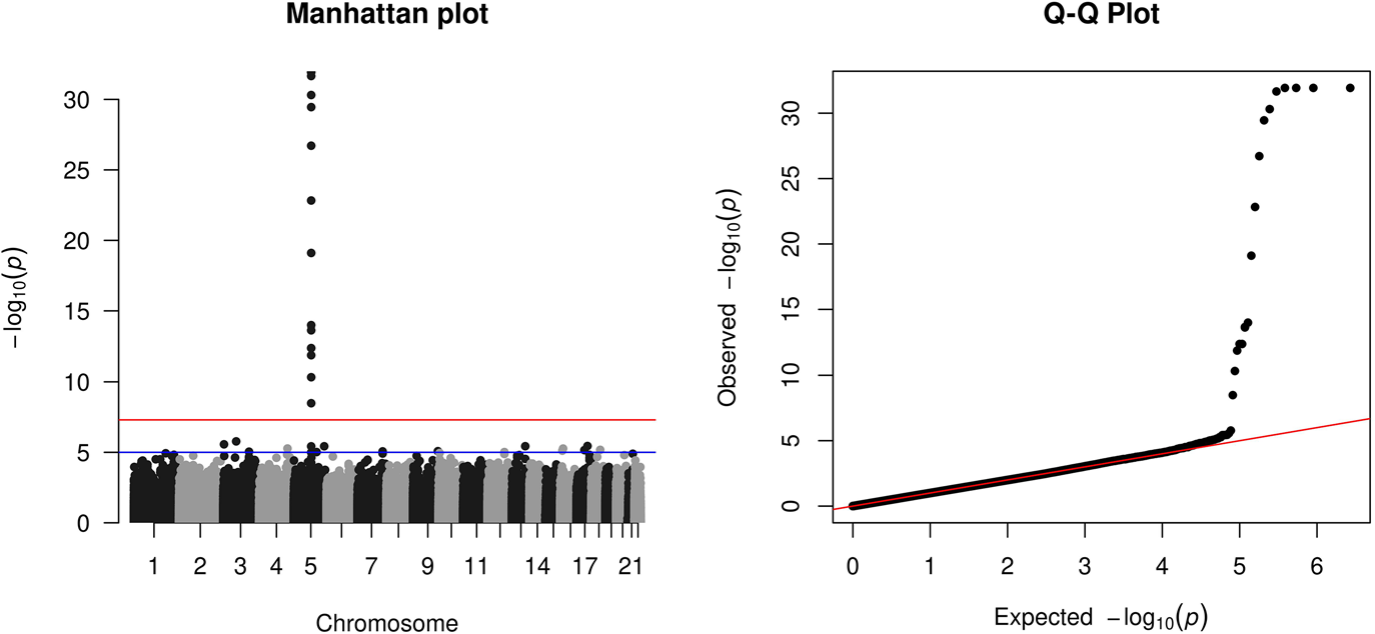
The Manhattan plot and Q-Q plot of the 1,344,840 p-values from the YRI GWAS of the gene expression of *ERAP2*, obtained using the workshop materials

Without going into the technical details, students are then introduced to the α = 5.0 × 10^−8^ genome-wide significance threshold used in GWAS to control the family wise error rate at 0.05 [20]. Further, two most commonly used data visualization plots in GWAS are introduced: the Manhattan plot and the Q-Q plot as shown in Fig. 2. These two plots complement the histogram which lumps all small p-values in one bin, thus masking the individual significant results.

The Q–Q plot in Fig. 2 is a standard statistical plot, showing the quantiles of the observed p-values against those of Unif(0,1), on the $-\text {log}\_{10}$ scale. In GWAS, the Q–Q plot serves two purposes. First, it highlights the significant results if there are any at the tail of the distribution. Second, it also shows the overall distribution of the GWAS p-values (though on the $-\text {log}\_{10}$ scale), which is typically expected to follow the main diagonal line.

Based on the Q–Q plot in Fig. 2, it is clear that several SNPs are significantly associated with the gene expression of *ERAP2* in the YRI GWAS. However, their genomic locations (e.g., from which chromosome) are unclear. Thus comes the Manhattan plot which contrasts the $-\text {log}\_{10}$ p-value of each SNP against its genomic location, with the $\alpha = 5.0 \times 10^{-8}$ genome-wide significance line (7.3 on $-\text {log}\_{10}$ scale) marked in red. Other significance thresholds for ‘suggestive’ association may also be shown, such as the $-\text {log}\_{10}(10^{-5})$ blue horizontal line included in Fig. 2.

In total, there are 17 genome-wide significant SNPs with p-values less than $5.0 \times 10^{-8}$, all from the locus on chromosome 5 (at 96.2 – 96.3 Mb) that is close to the *ERAP2* gene. These are called cis-eQTL SNPs, i.e., SNPs near the gene and whose genotypes associated with differences in the gene expression level.

Another noticeable feature in a typical Manhattan plot is the ‘clustering’ of significantly associated SNPs. This is due to the phenomenon called linkage disequilibrium (LD) between nearby SNPs [42]. The location, p-values, and the LD between SNPs of a significant locus may be visualized in a Manhattan-like plot using the LocusZoom service [3]. The implementation steps associated with the *ERAP2* example are included in the appended workshop manual. Although LD is akin to the statistical concept of correlation, it is an advanced concept in statistical genetics involving population genetics, thus not discussed further in this workshop.

### Summary of the GWAS Workshop Conducted

In the summer of 2021, our team offered this workshop to senior high school students from the University of Toronto Schools (UTS) in Toronto, Ontario, Canada. Due to the pandemic and limited number of TAs available, it was offered online and restricted to 15 participants, which were selected based on their interests and readiness in statistics, genetics and computing; see Appendix 1 for the application form. Post-workshop, a survey was conducted to collect participant feedback; see Appendix 1 for the survey questions.

Prior to the workshop, in addition to the survey, an earlier version of the appended manual was distributed to the participating students. Additionally, given the relatively low overall readiness of the participating students, the two lead TAs (AS and EE) provided detailed instructions for software installation and configuration, with a troubleshooting guide. Students followed this manual to work in groups, with clarification from the TAs via an online tutorial session as well as Discord discussion; Discord was the preferred social media of this group of students. At the time of the workshop, AS and EE were first year undergraduate students majored, respectively, in mathematics and life sciences, at the University of Toronto; AS and EE were mentored by ADP and LS during the summer of 2020.

Throughout the 4.5-day workshop, the morning lectures providing the necessary background in genetics and statistics were given, respectively, by ADP and LS. The afternoon sessions were guided tutorials, lead by AS and EE with participation of ADP and LS. Notably, on the last morning, students were encouraged to select a trait from the GWAS catalog^9^ [4] and to present a 3–5 min summary of a paper that performed a GWAS for that trait. In addition, students were encouraged to describe their motivation for selecting each particular trait, which provided an emotional connection to the science through personal stories, typically related to family history of diseases. The presented traits ranged from gout, breast cancer, to multiple sclerosis. Finally, to keep the students engaged, music were curated in advance and played during the (frequent) breaks, and the song “Another Brick in the Wall", by Pink Floyd, was much appreciated by the students based on their feedback.

## Student Feedback

After the workshop, a feedback survey (Appendix D) was distributed and eight responses were collected. Students found the workshop overall interesting, especially working with and interpreting the genetic component of the workshop. The students particularly enjoyed the SNP finding activity, and found the guided afternoon sessions helpful to their understanding.

Due to the high school background of the students, and the workshop’s limited time frame, some found the pace of the lectures to be overwhelming, particularly the statistical section of the lectures. Subsequently, notes were added to explain the difficult levels of the five lecture slide decks. Students unaccustomed to programming found using the terminal-based PLINK to be confusing, and recommended adding a terminal tutorial to the workshop manual, which was later included.

## Discussion

Depending on the experience of participants, the scope of the workshop may be extended, including covering more advanced lectures, analyses and plots, as well as analyzing additional datasets. Discussion around the cleaning of the 1000 Genomes data could be included in the morning lecture sessions, and cleaning steps for the 1000 Genomes individuals [40] may be replicated in the afternoons. More thorough descriptions of large-scale multiple testing and fundamentals of regression in the GWAS context may be included. An analysis using individuals with different populations, with PCA adjustment, may be given in the practical hands-on sessions. After conducting a sample GWAS in one population (e.g., the YRI GWAS), gene expressions with various significance [11] matched with other 1 KG populations may be provided for students to replicate. Included UNIX commands may be used as an introduction to conducting a remote GWAS on a cloud-based system, which typically are UNIX-based.

To adhere to the current standard of reproducible research [33], initial GWAS were conducted and documented independently by AS and EE. The two sets of results were then compared with each other, and the analyses and results were successfully reproduced, independently, by the workshop participants. Additionally, the observed *ERAP2* significance replicates the earlier work by Cheung et al. [11]. R, PLINK, and dataset versions were synchronized, and all scripts were version-controlled and hosted on the workshop GitHub. The exact analytical steps were recorded in a GWAS documentation, which would later become the appended, open source manual that allows users to reproduce the workshop GWAS materials. Finally, the tested workshop datasets and other materials were also made publicly available on Zenodo.^10^

## A Phenotype Extraction and Dataset Generation

Phenotype files containing gene expression data must be matched to cleaned, independent 1000 Genomes individuals to create the datasets. Please refer to Github.com/sugolov/GWAS-Workshop/Notebooks/DatasetPreparation.Rmd to create a phenotype file using the expression data from the University of Geneva Medical School [31, 43]. Phenotype files for ERAP2 are provided for the CEU and YRI populations for single population and mixed population analysis on Github.com/sugolov/GWAS-Workshop/Datasets. The PLINK binary format genotype files of independent samples were downloaded from http://tcag.ca/tools/1000genomes.html [40]. Refer to Github.com/sugolov/GWAS-Workshop/Notebooks/YRI_Analysis.Rmd to match the phenotype files with the 1 KG dataset from The Centre for Applied Genomics [2, 40]. For more information on required files and phenotype/genotype combination, please visit Sections 1.4 and 4.2 respectively of the manual, which can be found on the Github.

## B High Coverage Dataset

The High Coverage dataset was generated using the 30x High Coverage samples from the New York Genome Center (NYGC) [6]. Please refer to https://github.com/sugolov/GWAS-Workshop/tree/master/NotebooksGithub.com/sugolov/GWAS-Workshop/Notebooks/

High_Coverage.Rmd to generate a set of High Coverage data.

## C Application Form

The application form for students consisted of the following questions sent out as a Google Form.

1. Your name (First, Last)
2. Your email address
3. Please list relevant courses (UTS course codes and names) taken in statistics/data science, computer science and biology. (This is to help the workshop organizers to team up participants with complementing skills, if needed depending on the number of applicants.)
4. Check 1-2 boxes that reflect your strengths
  - Statistics/Data Science
  - Computing
  - Biology
5. Explain why are you particularly interested in this workshop? (200 words)
6. Any preference or suggestion on the platform(s) to be used for the virtual workshop, and for the on-line discussion board?
7. Any other comments?

## D End of Workshop Survey

The following questions were sent to the students as a Google Form after the end of the workshop. 8 students out of 17 responded.

1. On a scale of 1 to 10, how difficult did you find the genetic component?
2. If you answered greater than 7 to the question above please specify what you found too difficult. If you answered below 5 to the question please specify what you found too easy. If ≤ 5 your answer ≤ 7, still say something:-)
3. Did you find the pace of the genetic component too quick or too slow? Please specify.
4. What would you have liked to seen more of?
5. On a scale of 1 to 10, how difficult did you find the statistics component?
6. If you answered greater than 7 to the question above please specify what you found too difficult. If you answered below 5 to the question please specify what you found too easy. If ≤ 5 your answer ≤ 7, still say something:-)
7. Did you find the pace of the statistic component too quick or too slow? Please specify.
8. What would you have liked to seen more of?
9. On a scale of 1 to 10, how difficult did you find the computing/hands on component?
10. If you answered greater than 7 to the question above please specify what you found too difficult. If you answered below 5 to the question please specify what you found too easy. If ≤ 5 your answer ≤ 7, still say something:-)
11. Did you find the pace of the computing component too quick or too slow? Please specify.
12. What would you have liked to seen more of?
13. If we were to do this workshop again what would you have liked to see more of? Select all that apply.
  - Statistics
  - Genetics
  - Computing
  - Nothing. The balance was perfect.
  - Other:
14. What was your favorite aspect of the workshop? Select all that apply.
  - Statistics
  - Genetics
  - Computing
  - None. I did not enjoy anything
  - All. I loved everything
  - Other:
15. Would you like to have been presented with more references and resources before the workshop (i.e., terminal commands, file directory structure, etc)? If this is the case please specify.
16. From a scale of 1 - 10 how much did you enjoy the music during breaks?
17. Any other final remarks?

## Footnotes

1 https://www.internationalgenome.org/about

2 https://github.com/sugolov/GWAS-Workshop

3 https://github.com/LeiSunUofT/How-to-Run-a-GWAS.

4 https://tcag.ca/tools/1000genomes.html; https://www.internationalgenome.org/ were used for the genome-wide association study. We note that the 1000 Genomes Project data contains some family data, but the individuals used for this workshop are genetically unrelated to each other. This is to facilitate the application of a simple multivariate linear regression, as accounting for related individuals requires more advanced statistical knowledge; directly applying the simple regression to a dependent sample leads to increased false positive rates.

5 https://cran.r-project.org/doc/manuals/r-release/R-intro.pdf.

6 https://www.cog-genomics.org/plink/.

7 https://www.cog-genomics.org/plink/2.0/.

8 https://www.cog-genomics.org/plink/1.9/filter.

9 https://www.ebi.ac.uk/gwas/.

10 https://zenodo.org/record/7809150.
